# Genetic and Epigenetic Insights into Pregnancy-Related Complications

**DOI:** 10.3390/genes16010001

**Published:** 2024-12-24

**Authors:** Nihar R. Nayak, Akhilesh Srivastava, Manoj Kumar Jena, Anthony Odibo, Gary Sutkin

**Affiliations:** 1 Department of Obstetrics and Gynecology, University of Missouri-Kansas City School of Medicine, Kansas City, MO 64108, USA; aoodibo@umkc.edu (A.O.); sutking@umkc.edu (G.S.); 2 KCAS Bio, Olathe, KS 66061, USA; aksrivastava.aiims@gmail.com; 3 Department of Biotechnology, School of Bioengineering and Biosciences, Lovely Professional University, Phagwara 144411, Punjab, India; manoj.20283@lpu.co.in

Placental dysfunction is a leading cause of numerous pregnancy complications, including preeclampsia, preterm birth, fetal growth restrictions, placental abruption, and late spontaneous abortion. These conditions not only pose immediate risks to both maternal and fetal health but also have long-term implications for the well-being of both mothers and their offspring. Additionally, preexisting maternal conditions such as hypertension and diabetes, including their gestational forms, are strongly associated with placental abnormalities. Epigenetics, which involves molecular processes that regulate gene expression without altering the DNA sequence, plays a critical role in these conditions. Key epigenetic mechanisms, such as DNA methylation and histone modifications, are essential in regulating gene expression [1]. Recent studies have shown how these epigenetic modifications influence trophoblast cell differentiation and placental function, highlighting their importance in pregnancy outcomes. Understanding the genetic and epigenetic factors that contribute to placental disorders has become a central focus in the field of the developmental origins of health and disease (DOHaD).

Despite growing interest, the mechanisms underlying placental dysfunction remain poorly understood. However, recent advances in the field have shed new light on how maternal, placental, and fetal genetic and epigenetic regulations are interconnected and how these factors contribute to pregnancy complications and fetal developmental disorders. These discoveries not only enhance our understanding of the pathophysiology of these conditions but also offer promising avenues for developing novel biomarkers and therapeutic strategies. In this editorial, we highlight the original research and review articles featured in this Special Issue, which explore the complex genetic and epigenetic regulation involved in pregnancy complications and their potential clinical applications.

The advent of high-throughput sequencing technologies and their use in the analysis of differentially expressed genes have become central to identifying biomarkers for early diagnosis and developing predictive models for a range of diseases. In this Special Issue, Chou et al. (2021) present a thorough overview of the major advancements and technical challenges associated with high-throughput sequencing and differential gene expression data, focusing on quantifying fetal reprogramming in response to placental insufficiency and other intrauterine conditions, as well as predicting clinical outcomes [1]. Not only does placental insufficiency and fetal growth restriction impact the long-term health of the offspring, but emerging research also highlights the influence of maternal distress, pre-pregnancy health, and other pregnancy complications on the well-being of offspring. The authors propose that integrating cell-free fetal RNA (cffRNA) with high-throughput sequencing to study the dynamic transcriptional regulation involved in fetal reprogramming represents the most promising approach. They believe this method could lead to the development of clinically relevant predictive models, ultimately guiding therapeutic interventions for at-risk pregnancies.

Diabetes during pregnancy is associated with an increased risk of birth defects and pregnancy complications, such as fetal growth restriction (FGR) and preeclampsia, and also increases the likelihood of metabolic diseases in offspring later in life. In a study using a mouse model of type 1 diabetes, Kappen et al. (2022) explored potential candidate genes involved in placental growth and cellular abnormalities in diabetic pregnancies [2]. In a previous study using the same model, the authors found smaller placentas in which the sizes of the junctional zone and labyrinth were reduced. The current study focused specifically on genes associated with embryonic and placental growth, trophoblast invasion, cell migration, and inflammatory processes. By analyzing the expression of 47 candidate placental genes at different gestational stages in both normal and diabetic pregnancies, the authors linked the growth deficiencies in placentas and fetuses to impaired insulin growth factor (IGF2) and serotonin signaling pathways, as well as defective cell migration due to abnormal prostaglandin signaling. Additionally, the study highlighted that gene expression in the midgestation placenta (E10.5) is particularly sensitive to metabolic and nutritional disruptions, possibly reflecting the transition from histiotrophic to hemotrophic nutrition.

Failure of uterine spiral artery remodeling during pregnancy has been implicated in several pregnancy complications, including preeclampsia and FGR. Despite its crucial role in ensuring adequate placental blood flow, the mechanisms underlying spiral artery remodeling remain poorly understood. Genomic and proteomic studies on vascular tissues, particularly spiral arteries, are limited under both physiological and pathophysiological conditions. While mesenteric artery models have been widely used to study vascular responses to various stimuli, several studies suggest that the vascular responses of goat mesenteric arteries and human arteries are highly similar. In a study by Mohanty et al. (2022), the proteomic profile of acid stress-induced vasorelaxation was examined using a goat mesenteric artery model, combining classical pharmacology with cutting-edge proteomics [3]. By analyzing the vascular proteomes with 2D-GE, LC-MS/MS, and MALDI TOF MS, they identified several unique proteins, including actin, transgelin, WD repeat-containing protein 1, desmin, tropomyosin, ATP synthase β, Hsp27, aldehyde dehydrogenase, pyruvate kinase, and vitamin K epoxide reductase complex subunit 1-like protein. These findings offer new insights into potential adaptive mechanisms for maintaining vascular tone under acute stress, emphasizing the upregulation of actin proteoforms, actin-associated proteins, and mitochondrial ATP synthase. However, further research is needed to determine whether these adaptive mechanisms are also present in uterine arteries during both normal and complicated pregnancies.

Pregnancy complications, such as maternal intrauterine infections, preterm birth, preeclampsia, and systemic inflammation in both the mother and fetus, can lead to hypoxic–ischemic injury in the fetal brain. This type of injury is a major contributor to neurological disabilities and mortality in children worldwide. Hypoxic–ischemic damage affects specific regions of the developing brain at various stages, highlighting the importance of studying its impact on different cell types in the fetal and neonatal brain. While animal and in vitro studies suggest that glutamate excitotoxicity plays a central role in neuronal death following hypoxia, the molecular mechanisms and the involvement of various neural cells in glutamate excitotoxicity in humans remain poorly understood. To explore the role of astrocytes in hypoxic injury, Shrivastava et al. (2022) developed a novel culture model using a homogeneous population of primary human astrocytes derived from fetal neural stem cells (FNSCs) isolated from aborted fetal brain tissue [4]. Using this model, they specifically examined the effect of hypoxia on the expression and function of glutamate transporters in astrocytes. Their findings showed that differentiated astrocytes maintained both glutamate transporter expression and function, even under moderate and severe hypoxic conditions. Their novel in vitro model of human FNSC-derived astrocytes exposed to hypoxic injury not only supports the existing evidence of the relative resistance of astrocytes to hypoxic injury, but also strengthens this finding by showing that astrocytes exposed to hypoxia continue to maintain glutamate uptake. Collectively, these results suggest that human FNSC-derived astrocytes can maintain glutamate uptake following hypoxic injury, providing evidence for their potential neuroprotective role under hypoxic conditions.

Recurrent pregnancy loss (RPL) is a complex, multifactorial condition influenced by various genetic and environmental factors. Genetic causes, such as chromosomal abnormalities like aneuploidy, are thought to account for roughly 50% of RPL cases. Recent research has focused on identifying genetic markers linked to pregnancy loss, utilizing advanced molecular techniques such as chromosomal microarray analysis and next-generation sequencing. However, findings across studies have been inconsistent. While large copy-number variations (CNVs) are well-established as risk factors for miscarriage, there is a lack of large, systematic cohort studies specifically exploring the relationship between particular CNVs and RPL. Moreover, the presence of CNV differences between sporadic abortion (SA) and RPL cases remains unclear. Sheng and colleagues (2021) analyzed over 1500 miscarriage cases to investigate the role of embryonic chromosomal abnormalities and CNVs in RPL compared to SA [5]. Using a single-nucleotide polymorphism array (SNP-array) and CNV sequencing (CNV-seq), they identified chromosomal abnormalities in 57.52% of cases, with significant differences in the incidence and distribution of these abnormalities between the RPL and SA groups. The study also uncovered 346 CNVs in 173 cases, including 272 duplications and 74 deletions. Notably, duplications in regions 16q24.3 and 16p13.3 were more common in RPL cases, suggesting a potential association with the condition. Additionally, the study identified 213 genes and 131 signaling pathways as potential contributors to RPL. These findings highlight the significant role of CNVs in the pathogenesis of RPL, suggesting that a deeper understanding of the genetic mechanisms involved could lead to the development of a population-based diagnostic panel for identifying individuals at risk for RPL.

Drobek (2022) reviewed the fascinating and complex topic of paralogous genes in embryonic development, using the eye and other tissues as model systems [6]. The review presented compelling examples of how these genes functionally overlap during development, emphasizing the importance of gene dosage and its sensitivity, which can result in varying degrees of developmental disruption. Gene duplications, a natural consequence of evolution, lead to an increased gene dosage, with duplicated genes being either retained or eliminated through purifying selection. The review also addressed key aspects such as the extent of the functional equivalency retained by paralogous genes, their maintained redundancy, the gene dosage requirements, and the sensitivity of specific tissues, organs, and processes during development. Additionally, it discussed how duplicated genes acquire divergent functions over time. Alterations in gene dosage, or reductions below a critical threshold, can have significant phenotypic consequences, potentially leading to severe developmental defects or embryonic lethality.

As previously mentioned, the high incidence of adverse pregnancy outcomes, such as preeclampsia and FGR, continues to pose a significant challenge, with limited effective treatment options available. The majority of these complications are thought to stem from placental dysfunction, highlighting the need for innovative therapeutic strategies. However, a major obstacle in addressing these challenges is the difficulty in achieving the targeted delivery of treatments specifically to the placenta while minimizing potential risks to the fetus and avoiding systemic side effects. In their comprehensive review, Pepe and Albrecht (2021) provide valuable insights into recent advancements in placental drug and gene delivery technologies [7]. The review thoroughly discusses the current strategies, including the use of placental targeting peptide-bound liposomes, nanoparticle or adenoviral constructs decorated with specific peptide sequences, and placental gene promoters delivered via maternal intravenous injection, direct placental injection, or uterine artery infusion. It also discusses noninvasive, site-selective targeting methods, such as regulating genes conjugated with microbubbles for contrast-enhanced ultrasound. Furthermore, the review assesses the efficacy of these approaches in animal models and evaluates their potential for translation into human pregnancies complicated by placental dysfunction.

In conclusion, this Special Issue presents a comprehensive collection of new investigations and perspectives on the maternal, placental, and fetal genetic and epigenetic factors that contribute to pregnancy complications and fetal developmental disorders. It covers a broad array of topics, including high-throughput sequencing, differential gene expression and fetal reprogramming in response to placental insufficiency, the impact of diabetic pregnancies, hypoxic injury in the fetal brain, embryonic chromosomal abnormalities in recurrent pregnancy loss versus spontaneous abortion, paralogous genes in embryonic development, and recent advancements in placental drug and gene delivery technologies. While the progress made in understanding the genetic and epigenetic regulation of pregnancy complications associated with placental dysfunction and the developmental origins of health and disease is promising, it is clear that there is still much to be learned. Further research is needed to fully elucidate these complex processes in humans, which will ultimately pave the way for more effective treatments and interventions to improve maternal and fetal health outcomes.
